# Racial Differences in Triage for Emergency Department Patients with Subjective Chief Complaints

**DOI:** 10.5811/westjem.59044

**Published:** 2023-08-30

**Authors:** Cassandra Peitzman, Jossie A. Carreras Tartak, Margaret Samuels-Kalow, Ali Raja, Wendy L. Macias-Konstantopoulos

**Affiliations:** Massachusetts General Hospital, Harvard Medical School, Department of Emergency Medicine, Boston, Massachusetts

## Abstract

**Introduction:**

Black and Hispanic patients are frequently assigned lower acuity triage scores than White patients. This can lead to longer wait times, less aggressive care, and worse outcomes. In this study we aimed to determine whether these effects are more pronounced for patients with subjective complaints.

**Methods:**

We performed a retrospective analysis for all adult visits between 2016-2019 at an urban academic emergency department (ED) with acuity-based pods. We determined rates of initial high-acuity triage both across all patients and among the subset located in the high-acuity pod at time of disposition (either through initial assignment or subsequent up-triage). Analysis was performed for common chief complaints categorized as subjective (chest pain, dyspnea, any pain); observed (altered mental status); numeric (fever, hypotension); or protocolized (stroke, ST-elevation myocardial infarction). We constructed logistic regression models to control for age, race, gender, method of arrival, and final disposition.

**Results:**

We analyzed 297,355 adult ED visits. Black and Hispanic patients were less likely to be triaged to high-acuity beds (adjusted odds ratio [aOR] 0.76, 95% confidence interval [CI] 0.73-0.79 for Black, and aOR 0.87, 95% CI 0.84-0.90 for Hispanic patients). This effect was more pronounced for those with subjective chief complaints, including chest pain (aOR 0.76, 95% CI 0.73-0.79 for Black and 0.88, 95% CI 0.78-0.99 for Hispanic patients), dyspnea (aOR 0.79, 95% CI 0.68-0.92 and 0.8, 95% CI 0.72-0.99), and any pain (aOR 0.83, 95% CI 0.75-0.92 and 0.89, 95% CI 0.82-0.97, respectively). Among patients in the high-acuity pod at time of disposition, Black and Hispanic patients were disproportionately triaged to lower acuity pods on arrival (aOR 1.47, 95% CI 1.33-1.63 for Black and aOR 1.27, 95% CI 1.15-1.40 for Hispanic adults), with significant differences observed only for subjective chief complaints. No differences were observed for observed, objective, or protocolized complaints in either analysis.

**Conclusion:**

Black and Hispanic adults, including those who ultimately required high-acuity resources, were disproportionately triaged to lower acuity pods. This effect was more pronounced for patients with subjective chief complaints. Additional work is needed to identify and overcome potential bias in the assessment of patients with subjective chief complaints in ED triage.

Population Health Research Capsule What do we already know about this issue?

*Racial disparities in triage can lead to less aggressive care and worse outcomes.*

What was the research question?

*Is race-based triage more pronounced for patients with subjective chief complaints, such as pain and dyspnea?*

What was the major finding of the study?

*Black and Hispanic patients were less likely than similar White patients to be triaged to high-acuity bays when presenting with chest pain (aOR 0.76 for Black and 0.88 for Hispanic patients), dyspnea (aOR 0.79 and 0.80), or any pain (aOR 0.83 and 0.89). However, patients whose complaints activated protocolized pathways (e.g., “Code Stroke”) were triaged identically across racial groups.*

How does this improve population health?

*Further integration of objective data (eg, vital signs and ECGs) and protocols for specific complaints may help reduce disparities in triage.*

## INTRODUCTION

Over the past several decades, a robust literature has developed demonstrating racial-, gender-, and language-based disparities in the quality and intensity of medical care in the United States.^1^^–^^5^ Black and Hispanic patients are consistently offered less intensive care,^6^^–^^8^ subjected to longer wait times,^9^ and seen as less acutely ill then their White counterparts, even when controlling for other possible explanatory factors.^10^^,^^11^ In some cases, these differences can lead to delays in care, inadequate intensity of intervention or monitoring,^12^^–^^16^ and greater risk of adverse outcomes.^17^

Triage provides a natural context in which to assess encounter-level drivers of such disparities because of both its well-defined, episodic nature and because it initiates a treatment path that may influence a patient’s care throughout their clinical course. In this study we sought to 1) determine whether racial differences are present in either initial rates of high-acuity triage or need for later re-assignment to a high-acuity pod and 2) whether these differences vary by patient chief complaint. We hypothesized that Black and Hispanic patients experience higher rates of under-triage, and these differences are more pronounced for patients presenting with subjective or symptom-based chief complaints. This hypothesis is in keeping with prior literature suggesting that subjective assessments with incomplete information may lead to greater introduction of bias,^18^ whereas chief complaints that trigger clear protocols (such as ST-elevation myocardial infarction [STEMI] or stroke alerts) may tend toward more prescriptive and, therefore, less biased triage processes. We hope that by identifying the circumstances under which racial disparities in triage appear, we may better understand and thereby intervene and act upon the phenomena that drive them.

## METHODS

We conducted a retrospective analysis of all adult patient visits between 2016–2019 to an urban academic ED with nursing-led triage to acuity-based pods (including low-acuity/fast-track, mid-acuity, and critical-care/high-acuity pods) based on hospital-specific, resource-based guidelines. Our analysis considered both the full set of visits and selected chief complaints, which were chosen to represent four types of complaint: “subjective” complaints were those relating to patients’ reports of their own symptoms; “objective” complaints were defined by numeric cutoffs in prehospital or home assessments; “observed” complaints were subjectively defined but reported based on assessments by a third party; and finally, “protocolized” chief complaints were defined as those for which triage is assigned by protocol.

For this, we included the three most common chief complaints with at least a 20% rate of high-acuity triage (chest pain and shortness of breath as “subjective” complaints and altered mental status as “observed”). “Objective” complaints included both the most common and highest acuity complaints with numerical definitions (fever and hypotension). Two common “protocolized” chief complaints (STEMI and stroke) were also included. To better assess a broad group of subjective complaints, we assessed an additional category of any chief complaint including “pain,” (approximately 10% of which was initially triaged as high acuity). Chief complaints were identified via search and manual review of free-text chief complaints entered at triage. Racial categories were taken from data entered at time of registration, with pooled categories including Black, White, Asian, multiracial, other, and unknown. Records with missing variables (316 total) were excluded from the analysis.

We evaluated two outcomes of interest: relative probability of initial triage to the high-acuity pod (Table 2a) and relative probability of having required up-triage (reassignment to the high-acuity pod) for patients ultimately requiring high-acuity care (Table 2b). Logistic regression was performed to assess the relationship between these outcome variables and self-reported race, across both the full sample and by chief complaint. Controls were included for gender, age (including squared and bin terms), method of arrival (ambulance vs walk-in), and final disposition (admission, observation, discharge, or death). We performed analysis was performed in R 4.1.0 (R Foundation for Statistical Computing, Vienna, Austria),^19^^,^^20^ with results reported as odds ratios for ease of interpretation. Although moderate collinearity was identified among our control variables, variance inflation factors were <2 in all cases, and main effects were robust to multiple model specifications. (See Appendix 1 for representative sensitivity analyses.) The study was reviewed and approved by the hospital Institutional Ethics Review Board.

**Table 1.** Summarized racial, gender, and age distribution of full adult emergency department sample 2016–2019.

	Mean age (years)	Percentage male	Percentage high-acuity triage	Number
White	50.7	52.9%	19%	210,596
Black	42.4	52.7%	11.7%	32,645
Hispanic	34.0	49.9%	10.6%	49,973
Asian	41.5	47.1%	14.3%	14,875
Multiracial	41.48	51.5%	14.6%	8,216
Other	37.7	51.4%	11%	10,833
Unknown	39.4	53.7%	20.1%	7,154

**Table 2.** (A) Adjusted odds of initial triage to high-acuity pod and (B) adjusted odds of initial lower-acuity triage among patients completing emergency department (ED) course in high-acuity pod among adult ED patients 2016–2019, stratified by chief complaint. Controls included for age, age squared, age categories (18–44 years, 45–64 years, 65+ years), ED death and admission. “Other” and “Unknown” racial categories omitted for clarity. STEMI and stroke-alert patients were uniformly triaged to a high-acuity setting and, therefore, regression analysis was not possible. Results reported as adjusted odds ratios with 95% confidence intervals.

2A. Adjusted odds of triage to high-acuity pod by race								
Chief complaint	All patients	Chest pain	Dyspnea	Pain	Fever	Hypotension	AMS
Black	0.76^***^	0.77^***^	0.79^**^	0.83^***^	1.08	0.99	1.06
	(0.73, 0.79)	(0.67, 0.88)	(0.68, 0.92)	(0.75, 0.92)	(0.85, 1.37)	(0.70, 1.41)	(0.44, 2.54)
Hispanic	0.87^***^	0.88^*^	0.84^*^	0.89^**^	1	0.99	1.07
	(0.84, 0.90)	(0.78, 0.99)	(0.72, 0.99)	(0.82, 0.97)	(0.77, 1.30)	(0.76, 1.29)	(0.46, 2.51)
Asian	1.06^*^	1.07	1.15	1.13	1.24	1.33^*^	2.63
	(1.01, 1.12)	(0.88, 1.30)	(0.92, 1.44)	(0.99, 1.30)	(0.84, 1.82)	(1.01, 1.75)	(0.74, 9.30)
Multiracial	0.91^*^	1.15	0.9	1.05	0.69	1.05	2.32
	(0.85, 0.98)	(0.88, 1.50)	(0.68, 1.20)	(0.88, 1.26)	(0.44, 1.06)	(0.62, 1.78)	(0.27, 20.20)
Gender (male)	1.26^***^	1.55^***^	1.27^***^	1.58^***^	1.03	1.21^*^	1.23
	(1.23, 1.28)	(1.43, 1.68)	(1.18, 1.38)	(1.50, 1.67)	(0.91, 1.18)	(1.04, 1.40)	(0.82, 1.84)
BIBAª	3.01^***^	2.66^***^	1.78^***^	2.59^***^	2.16^***^	2.39^***^	1.80^**^
	(2.95, 3.07)	(2.45, 2.88)	(1.64, 1.93)	(2.45, 2.73)	(1.88, 2.48)	(2.04, 2.81)	(1.20, 2.69)
Observations	297,034	16,171	13,150	73,486	4,108	6,331	638
2B. Adjusted odds of initial lower-acuity triage for patients requiring high-acuity resources prior to disposition by race								
Chief complaint	All patients	Chest pain	Dyspnea	Pain	Fever	Hypotension	AMS
Black	1.47^***^	1.68^***^	1.3	1.47^***^	1.15	0	1.28
	(1.33, 1.63)	(1.25, 2.26)	(0.90, 1.89)	(1.18, 1.83)	(0.49, 2.70)	(0.00, Inf)	(0.64, 2.55)
Hispanic	1.27^***^	1.08	1.54^*^	1.11	1.34	1.28	1.17
	(1.15, 1.40)	(0.79, 1.47)	(1.06, 2.24)	(0.90, 1.37)	(0.72, 2.48)	(0.12, 13.10)	(0.52, 2.63)
Asian	1.09	1.09	1.01	1.15	1.23	2.3	1.52
	(0.94, 1.26)	(0.70, 1.72)	(0.57, 1.79)	(0.85, 1.56)	(0.65, 2.35)	(0.26, 20.40)	(0.53, 4.37)
Multiracial	1.11	1.64	1	1.59^*^	1.68	0	2.06
	(0.91, 1.36)	(0.98, 2.77)	(0.45, 2.19)	(1.08, 2.35)	(0.53, 5.26)	(0.00, Inf)	(0.70, 6.05)
Gender (male)	0.91^**^	1.03	0.77^*^	0.91	0.94	0.35	1.34
	(0.86, 0.96)	(0.85, 1.25)	(0.63, 0.95)	(0.80, 1.03)	(0.66, 1.35)	(0.08, 1.48)	(0.90, 2.02)	
BIBAª	0.65^***^	0.52^***^	0.87	0.65^***^	0.84	0.62	0.61^*^
	(0.61, 0.69)	(0.43, 0.63)	(0.71, 1.07)	(0.57, 0.74)	(0.57, 1.23)	(0.15, 2.52)	(0.40, 0.94)
Observations	51,902	5,535	4,564	7,845	932	419	1,895

* *P* < 0.05; **P < 0.01; ***P < 0.001.

*AMS*, altered mental status; *BIBA*, brought in by ambulance.

## RESULTS

Of 297,355 adult ED visits analyzed, 66% (196,040) were of patients who identified as White, approximately 10% (29,214) who identified as Black, and 13% (38,396) who identified as Hispanic. Patients were 48% (143,079) female, 52% (154,268) male, and had a mean age of 51 years.

Overall, the adjusted odds of triage to the high acuity pod were lower for Black (adjusted odds ratio [aOR] 0.76, 95% confidence interval [CI] 0.73-0.79 and Hispanic patients aOR 0.87, 95% CI 0.84-0.90). Among our identified chief complaints, this effect was only demonstrated for patients with subjective chief complaints, including chest pain (aOR 0.76, 95% CI 0.73, 0.79 for Black, and aOR 0.88, 95% CI 0.78, 0.99 for Hispanic patients), dyspnea (aOR 0.79, 95% CI 0.68-0.92 for Black, and aOR 0.84, 95% CI 0.72-0.99 for Hispanic patients), and any pain (aOR 0.83, 95% CI 0.75-0.92 for Black, and aOR 0.89, 95% CI 0.82-0.97 for Hispanic patients). No differences were detected across observed, numeric, or protocolized complaints.

We performed analysis of need for up-triage on the subset of patients located in the high-acuity pod at time of ED disposition (death, hospital admission, or discharge), constituting approximately 16% of adult visits (51,959). Patients were considered to have required up-triage if they were initially assigned to a lower acuity pod and required reassignment to the high-acuity pod during their ED course. Racial differences were also identified in this measure, with Black and Hispanic adults experiencing higher rates of up-triage. This was demonstrated across the full all-complaint study sample (aOR of 1.47, 95% CI 1.3-1.63 for Black, and aOR of 1.27, 95% CI 1.15-1.40 for Hispanic adults), as well as for Black patients presenting with chest pain (aOR 1.68, 95% CI 1.25-2.26), or any pain (aOR 1.47, 95% CI 1.1-1.83) and Hispanic patients presenting with dyspnea/shortness of breath (aOR 1.54, 95% CI 1.06-2.24). No differences were observed for observed, objective, or protocolized complaints.

## DISCUSSION

In our analysis we found that Black and Hispanic adults in our population were disproportionately triaged to lower acuity areas, and that this phenomenon was more pronounced for patients presenting with subjective chief complaints. Further analyses demonstrated that of patients requiring critical care/high-acuity resources at the time of ED discharge, Black and Hispanic patients tended to have been disproportionately triaged to lower acuity pods during initial assessment. These findings suggest that the pattern of lower acuity triage cannot be explained by true differences in resource requirements over the ED course (ie, accurate prediction of lower resource requirements related to less severe clinical presentations), but rather a tendency to consistently underestimate the needs of Black and Hispanic adults. This pattern is also more pronounced for patients presenting with subjective chief complaints, suggesting that triage clinicians’ assessments of the severity of patient-reported symptoms for Black and Hispanic patients may have played a role in this underestimation.

Many potential mechanisms may underlie this pattern, possibly including racially correlated differences in patients’ descriptions of their symptoms,^21^^,^^22^ differences in affective communication and stoicism,^23^ differences in symptom presentation from “canonical” cases historically used in medical education,^24^^,^^25^ differences in style or content of report or in actions taken by prehospital personnel,^26^ differential impact of clinicians’ cognitive “heuristics” regarding disease presentation,^27^^–^^29^ and differences in patient-clinician interaction style or other forms of bias.^30^^–^^32^ These phenomena may also have been exacerbated by structural factors (such as ease of access to interpreter services when needed, crowding, clinician fatigue or cognitive burden, time of day, etc), which are beyond the scope of our analysis. Reassuringly, we did not observe racially correlated triage differences in protocolized chief complaints.

## LIMITATIONS

This was a single-center study that used an acuity-based triage system to identify race-related differences in triage assignment, potentially limiting generalizability of this finding. This analysis also focused on a subset of ED chief complaints that represent approximately 32% of total ED presentations and were developed based on frequency, acuity, and ease of identification in our data. It is possible that these patterns would not emerge in a dataset where other chief complaints were more common, more frequently represented high-acuity presentations, or were more readily identifiable. This analysis was also performed on data collected under hospital-developed triage guidelines but prior to the 2021-2022 implementation of a formalized Emergency Severity Index (ESI) assignment protocol within our system, which may alter these patterns.

In addition to the potential structural factors listed above, we did not control for other interpersonal or individual factors that may contribute to pod selection within this system (including current staffing, hourly throughput time, relative crowding, recent triage to the same pod, etc). Neither did we assess nursing factors (including race, age, seniority, languages spoken, etc.). Thus, further work will be needed to both assess these additional factors and to identify potential mechanisms underlying our findings.

## CONCLUSION

Overall, our analysis identifies a pattern of significant racial differences in triage accuracy, which tends to underestimate the critical-care needs of Black and Hispanic adults, especially those with symptom-based complaints, potentially compromising both the timeliness and appropriateness of their care. These findings suggest that further work to better understand and improve triage encounters and the nature of the interactions within them may be important in helping to reduce disparities in ED care.

## Supplementary Information


