# Supplementary material for: Racial Differences in Triage for Emergency Department Patients with Subjective Chief Complaints
Source: West J Emerg Med. 2023 Aug 30;24(5):888–93. doi: 10.5811/westjem.59044 (PMC10527826; doi:10.5811/westjem.59044)
Supplement: Supplementary file 1 [file wjem-24-888-s001.docx]

Appendix 1: Representative Sensitivity Analyses with Primary Outcome Observed Across Multiple Model Specifications

(A) Odds of initial triage to High -Acuity Pod and (B) Odds of initial lower-acuity triage among patients completing ED course in high-acuity pod among adult ED patients 2016-2019. Models include controls for Age, Age Squared, Age Categories (18-44 years, 45-64 years, 65+ years), ED Death and Admission with results of multiple specifications shown below. “Other” and “Unknown” Racial categories omitted for clarity.

|  | 1a: Adjusted Odds of High-Acuity Initial Triage Across All Patients | | | | | | | | | | | | |
| --- | --- | --- | --- | --- | --- | --- | --- | --- | --- | --- | --- | --- | --- |
|  |  | | *Model 1* | | *Model 2* | | *Model 3* | | *Model 4* | | *Model 5* | | *Model 6* |
|  |  | |  | |  | |  | |  | |  | |  |
| Race | Black | | 0.57**** | | 0.57**** | | 0.71**** | | 0.71**** | | 0.69**** | | **0.76****** |
|  |  | | (0.55, 0.59) | | (0.55, 0.59) | | (0.69, 0.74) | | (0.69, 0.74) | | (0.66, 0.72) | | (0.73, 0.79) |
|  |  | |  | |  | |  | |  | |  | |  |
|  | Hispanic | | 0.57**** | | 0.60**** | | 0.81**** | | 0.82**** | | 0.81**** | | **0.87****** |
|  |  | | (0.55, 0.59) | | (0.58, 0.62) | | (0.79, 0.84) | | (0.79, 0.85) | | (0.78, 0.84) | | (0.84, 0.90) |
|  |  | |  | |  | |  | |  | |  | |  |
|  | Asian | | 0.73**** | | 0.88**** | | 0.89**** | | 0.89**** | | 1.02 | | **1.06*** |
|  |  | | (0.70, 0.77) | | (0.84, 0.93) | | (0.85, 0.94) | | (0.85, 0.94) | | (0.97, 1.08) | | (1.01, 1.12) |
|  |  | |  | |  | |  | |  | |  | |  |
|  | Multiracial | | 0.78**** | | 0.78**** | | 0.90** | | 0.91** | | 0.89*** | | 0.91* |
|  |  | | (0.73, 0.83) | | (0.73, 0.84) | | (0.84, 0.96) | | (0.85, 0.97) | | (0.83, 0.95) | | (0.85, 0.98) |
|  |  | |  | |  | |  | |  | |  | |  |
| Age Variables | Age (Years) | |  | |  | | 1.03**** | | 1.03**** | | 1.03**** | | 1.02**** |
|  |  | |  | |  | | (1.03, 1.03) | | (1.03, 1.04) | | (1.03, 1.04) | | (1.02, 1.03) |
|  |  | |  | |  | |  | |  | |  | |  |
|  | Age Squared | |  | |  | |  | | 1.00**** | | 1.00**** | | 1.00**** |
|  |  | |  | |  | |  | | (1.00, 1.00) | | (1.00, 1.00) | | (1.00, 1.00) |
|  |  | |  | |  | |  | |  | |  | |  |
|  | Age Bin (45-64) | |  | |  | |  | | 1.14**** | | 1.12**** | | 1.11*** |
|  |  | |  | |  | |  | | (1.09, 1.20) | | (1.06, 1.18) | | (1.05, 1.17) |
|  |  | |  | |  | |  | |  | |  | |  |
|  | Age Bin (65-94) | |  | |  | |  | | 1.33**** | | 1.41**** | | 1.30**** |
|  |  | |  | |  | |  | | (1.24, 1.43) | | (1.31, 1.52) | | (1.21, 1.40) |
|  |  | |  | |  | |  | |  | |  | |  |
|  | Age Bin (95+) | |  | |  | |  | | 1.14 | | 1.11 | | 1.06 |
|  |  | |  | |  | |  | | (0.99, 1.30) | | (0.97, 1.28) | | (0.92, 1.23) |
|  |  | |  | |  | |  | |  | |  | |  |
| Male | | |  | | 1.26**** | |  | |  | | 1.31**** | | **1.26****** |
|  |  |  |  | | (1.23, 1.28) | |  | |  | | (1.28, 1.34) | | (1.23, 1.28) |
|  |  | |  | |  | |  | |  | |  | |  |
| BIBA* | | |  | | 4.25**** | |  | |  | | 3.54**** | | **3.01****** |
|  |  |  |  | | (4.16, 4.33) | |  | |  | | (3.47, 3.61) | | (2.95, 3.07) |
|  |  | |  | |  | |  | |  | |  | |  |
| Expired | | |  | |  | |  | |  | |  | | 115.53**** |
|  |  |  |  | |  | |  | |  | |  | | (63.00, 212.00) |
|  |  | |  | |  | |  | |  | |  | |  |
| Admitted | | |  | |  | |  | |  | |  | | 4.53**** |
|  |  |  |  | |  | |  | |  | |  | | (4.43, 4.63) |
|  |  | |  | |  | |  | |  | |  | |  |
| Constant | | | 0.25**** | | 0.12**** | | 0.04**** | | 0.04**** | | 0.03**** | | 0.02**** |
|  |  |  | (0.25, 0.25) | | (0.12, 0.12) | | (0.04, 0.05) | | (0.04, 0.05) | | (0.02, 0.03) | | (0.02, 0.03) |
|  |  | |  | |  | |  | |  | |  | |  |
| Observations | | | 297128 | | 297128 | | 297128 | | 297034 | | 297034 | | 297034 |
| Log Likelihood | | | -138417.2 | | -127465.4 | | -131043.1 | | -130880.7 | | -122987.2 | | -113260.6 |
| Akaike Inf. Crit. | | | 276848.4 | | 254948.9 | | 262102.3 | | 261785.5 | | 246002.5 | | 226553.1 |
| *^*^Brought in By Ambulance* | | | | |  | | *p< 0.05; **p<0.01; ***p<0.001 | | | | | | |
|  | |  | |  | |  | |  | |  | |  | |

|  | 1b: Adjusted Odds of Non-Acute Initial Triage for Patients Requiring Acute Care Prior to Disposition | | | | | |
| --- | --- | --- | --- | --- | --- | --- |
|  |  | *Model 1* | *Model 2* | *Model 3* | *Model 4* | *Model 5* |
|  |  |  |  |  |  |  |
| Race | Black | 1.43**** | 1.45**** | 1.41**** | 1.44**** | **1.47****** |
|  |  | (1.29, 1.58) | (1.31, 1.61) | (1.27, 1.56) | (1.30, 1.60) | (1.33, 1.63) |
|  |  |  |  |  |  |  |
|  | Hispanic | 1.22**** | 1.25**** | 1.20*** | 1.24**** | **1.27****** |
|  |  | (1.11, 1.34) | (1.14, 1.37) | (1.09, 1.32) | (1.13, 1.37) | (1.15, 1.40) |
|  |  |  |  |  |  |  |
|  | Asian | 1.13 | 1.09 | 1.13 | 1.09 | 1.09 |
|  |  | (0.98, 1.31) | (0.94, 1.26) | (0.98, 1.30) | (0.94, 1.26) | (0.94, 1.26) |
|  |  |  |  |  |  |  |
|  | Multiracial | 1.08 | 1.11 | 1.08 | 1.11 | 1.11 |
|  |  | (0.89, 1.32) | (0.91, 1.35) | (0.89, 1.32) | (0.91, 1.35) | (0.91, 1.36) |
|  |  |  |  |  |  |  |
|  |  |  |  |  |  |  |
| Age Variables | Age (Years) |  |  | 1.01 | 1.01 | 1.00 |
|  |  |  |  | (1.00, 1.02) | (0.99, 1.02) | (0.99, 1.02) |
|  |  |  |  |  |  |  |
|  | Age Squared |  |  | 1.00 | 1.00 | 1.00 |
|  |  |  |  | (1.00, 1.00) | (1.00, 1.00) | (1.00, 1.00) |
|  |  |  |  |  |  |  |
|  | Age Bin (45-64) |  |  | 1.00 | 1.02 | 1.01 |
|  |  |  |  | (0.86, 1.17) | (0.87, 1.19) | (0.86, 1.18) |
|  |  |  |  |  |  |  |
|  | Age Bin (65-94) |  |  | 0.99 | 0.98 | 0.97 |
|  |  |  |  | (0.80, 1.22) | (0.80, 1.21) | (0.79, 1.19) |
|  |  |  |  |  |  |  |
|  | Age Bin (95+) |  |  | 1.02 | 1.03 | 1.02 |
|  |  |  |  | (0.69, 1.51) | (0.69, 1.53) | (0.69, 1.52) |
|  |  |  |  |  |  |  |
| Male | |  | 0.94* |  | 0.93* | **0.91**** |
|  |  |  | (0.88, 0.99) |  | (0.88, 0.99) | (0.86, 0.96) |
|  |  |  |  |  |  |  |
| BIBA* | |  | 0.67**** |  | 0.67**** | 0.65**** |
|  |  |  | (0.63, 0.70) |  | (0.63, 0.71) | (0.61, 0.69) |
|  |  |  |  |  |  |  |
| Expired | |  |  |  |  | 0.49* |
|  |  |  |  |  |  | (0.26, 0.92) |
|  |  |  |  |  |  |  |
| Admitted | |  |  |  |  | 1.55**** |
|  |  |  |  |  |  | (1.46, 1.65) |
|  |  |  |  |  |  |  |
| Constant | | 0.11**** | 0.14**** | 0.09**** | 0.12**** | 0.11**** |
|  |  | (0.10, 0.11) | (0.13, 0.15) | (0.06, 0.12) | (0.09, 0.17) | (0.08, 0.16) |
|  |  |  |  |  |  |  |
| Observations | | 51,930 | 51,930 | 51,902 | 51,902 | 51,902 |
| Log Likelihood | | -17,054.01 | -16,954.43 | -17,047.17 | -16,950.24 | -16,842.25 |
| Akaike Inf. Crit. | | 34,122.02 | 33,926.86 | 34,118.35 | 33,928.48 | 33,716.51 |
| *^*^Brought in By Ambulance* | |  |  | *p< 0.05; **p<0.01; ***p<0.001 | | |
